# Symptoms of postpartum anxiety and depression among women in Canada: findings from a national cross-sectional survey

**DOI:** 10.17269/s41997-020-00420-4

**Published:** 2020-10-20

**Authors:** Mihaela Gheorghe, Mélanie Varin, Suzy L. Wong, Melissa Baker, Vera Grywacheski, Heather Orpana

**Affiliations:** 1 Public Health Agency of Canada, 785 Carling Avenue, Ottawa, ON K1S 5H4 Canada; 2 School of Epidemiology and Public Health, University of Ottawa, Alta Vista Campus, Room 101, 600 Peter Morand Crescent, Ottawa, ON K1G 5Z3 Canada; 3 Royal Ottawa Institute for Mental Health Research, 1145 Carling Ave, Ottawa, ON K1Z 7K4 Canada

**Keywords:** Maternal health, Mental health, Postpartum depression, Postpartum anxiety disorder, Pregnancy, Santé maternelle, santé mentale, dépression post-partum, anxiété post-partum, grossesse

## Abstract

**Objective:**

This study presents national estimates on symptoms consistent with postpartum anxiety (PPA) and postpartum depression (PPD) and the association between these conditions and possible risk and protective factors in women who gave birth in Canada.

**Methods:**

Data were collected through the Survey on Maternal Health, a cross-sectional survey administered in Canada’s ten provinces between November 2018 and February 2019 among women who gave birth between January 1 and June 30, 2018. A total of 6558 respondents were included. Weighted prevalence estimates were calculated, and logistic regression was used to model the relationship between symptoms consistent with PPA, PPD, and potential risk factors.

**Results:**

Overall, 13.8% of women had symptoms consistent with PPA, while the prevalence of having symptoms consistent with PPD was 17.9%. Results of the logistic regression models indicated that women who had a history of depression were 3.4 times (95% CI 2.7–4.2) more likely to experience symptoms consistent with PPA and 2.6 times more likely to experience symptoms consistent with PPD (95% CI 2.2–3.2) compared with those who did not. Women who reported good, fair, or poor physical health were 2.4 times more likely to experience symptoms consistent with PPD (95% CI 2.0–2.9) and 2.0 times more likely to experience symptoms consistent with PPA (95% CI 1.7–2.4) compared with those who reported very good or excellent health. Maternal marital status, other postpartum maternal support, and sense of community belonging were also significant.

**Conclusion:**

This study highlights that a history of depression and good, fair, or poor physical health are associated with an increased odds of symptoms consistent with PPA and PPD, while other maternal support and sense of community belonging are associated with a decreased odds of these conditions.

## Résumé

**Objectif:**

La présente étude présente des estimations nationales sur les symptômes correspondant à de l’anxiété post-partum (APP) et à une dépression post-partum (DPP) et sur l’association entre ces affections et les éventuels facteurs de risque et de protection chez les femmes ayant accouché au Canada.

**Méthodologie:**

Les données ont été recueillies dans le cadre de l’Enquête sur la santé maternelle, une enquête transversale menée dans les dix provinces canadiennes entre novembre 2018 et février 2019 auprès des femmes ayant accouché entre le 1^er^ janvier et le 30 juin 2018. Au total, 6 558 personnes ont répondu à l’enquête. Des estimations de la prévalence pondérée ont été calculées, et une régression logistique a été utilisée pour modéliser la relation entre les symptômes correspondant à de l’APP, à une DPP et aux facteurs de risque possibles.

**Résultats:**

Dans l’ensemble, 13,8 % des femmes présentaient des symptômes compatibles avec de l’APP, tandis que la prévalence des symptômes compatibles avec une DPP était de 17,9 %. Les résultats des modèles de régression logistique ont indiqué que les femmes ayant des antécédents de dépression étaient 3,4 fois (IC à 95 % : 2,7 à 4,2) plus susceptibles de présenter des symptômes compatibles avec de l’APP et 2,6 fois plus susceptibles de présenter des symptômes compatibles avec une DPP (IC à 95 % : 2,2 à 3,2) par rapport à celles qui n’en avaient pas. Les femmes qui ont déclaré être en bonne, moyenne ou mauvaise santé physique étaient 2,4 fois plus susceptibles d’éprouver des symptômes correspondant à une DPP (IC à 95 % : 2,0 à 2,9) et 2,0 fois plus susceptibles d’éprouver des symptômes correspondant à de l’APP (IC à 95 % : 1,7 à 2,4) par rapport à celles qui ont déclaré être en très bonne ou en excellente santé. L’état matrimonial de la mère, les autres formes de soutien maternel post-partum et le sentiment d’appartenance à la collectivité étaient également significatifs.

**Conclusion:**

Cette étude souligne que des antécédents de dépression et une bonne, moyenne ou mauvaise santé physique sont associés à une augmentation de la probabilité de symptômes correspondant à de l’APP et à une DPP, tandis que d’autres formes de soutien maternel et le sentiment d’appartenance à la collectivité sont associés à une diminution de la probabilité de ces affections.

## Introduction

Following childbirth, mothers may experience a range of emotions, including deep sadness or worrying, which can appear within days and last for years if left undiagnosed (Lanes et al. 2011; Pawluski et al. 2017). Approximately 6–13% of women in developed countries report symptoms consistent with postpartum depression (PPD), while estimates of postpartum anxiety (PPA) range more widely, from 13 to 40% (Field 2018). More recently, Shoreya et al. (2018) estimated the prevalence of PPD at 17% globally. These conditions often remain undiagnosed and untreated in part because some of the associated symptoms, such as fatigue, weight fluctuation, or sleep disturbance, may be expected during the postpartum period (Iliadis et al. 2018). In addition to these more generalized symptoms, PPA may also be characterized by excessive worrying and obsessing, while PPD manifests as long-lasting and potentially severe feelings of sadness and hopelessness (Iliadis et al. 2018). PPD is often comorbid with PPA and symptomology is associated with greater severity (Pawluski et al. 2017; Ramakrishna et al. 2019). The strongest predictors of PPD include: depression during pregnancy, anxiety during pregnancy, experiencing stressful life events during pregnancy or the early puerperium, low levels of social support, and a previous history of depression (Robertson et al. 2004).

PPD may have immediate effects on the mother, such as impaired ability to cope with life events and parenting tasks (Brummelte and Galea 2016). The condition may also have a negative impact on the mother-infant attachment, sensitivity to her child, and family functioning, which can create lasting effects on the child’s brain development and reaction to stress (Brummelte and Galea 2016). Similarly, PPD may affect the health of other children in the family and the partner relationship.

The last national survey to measure PPD in Canada was the Maternity Experiences Survey, which was conducted in 2006 (PHAC 2009). There are currently no national estimates of symptoms consistent with PPA. The main objectives of this study were to present the first national estimates on symptoms consistent with postpartum anxiety, to provide updated estimates of symptoms consistent with postpartum depression, and to describe characteristics of women who gave birth in Canada between January 1 and June 30, 2018. The secondary objective of this study was to report on the association between these conditions and possible risks and protective factors during pregnancy and postpartum.

## Methods

Data were extracted from the Survey on Maternal Health (SMH), which was administered from November 29, 2018 to February 5, 2019. The Child Tax Benefit registry was used as the sampling frame to identify women who had given birth between January 1 and June 30, 2018, and included women five to thirteen months postpartum. The target sample included 13,000 dwellings in the ten provinces. Data were collected from 7085 women (54.7% response rate). Of these women, 6558 (92.6%) agreed to share their responses with the Public Health Agency of Canada (PHAC). All women who agreed to share their responses were included in the study. Of these, 4676 completed an electronic questionnaire and 2409 completed the survey by computer-assisted telephone interview. To ensure confidentiality and protection of privacy, data collected in this survey were under the authority of the Statistics Act.

### Survey content

Symptoms consistent with postpartum depression were measured through the 5-item version of the Edinburgh Postpartum Depression Scale (EPDS-5) (Eberhard-Gran et al. 2007). Women who scored 7 or above were considered to have a moderate symptom level of PPD in the seven days prior to completing the survey. The EPDS-5 correlates well with the full EPDS (*r* = 0.96) and demonstrated a Cronbach’s alpha of 0.76. Symptoms consistent with generalized anxiety symptoms were measured through the 2-item Generalized Anxiety Disorder scale (GAD-2) (Nath et al. 2018; Kroenke et al. 2007). The GAD-2 has demonstrated a sensitivity of 69% and a specificity of 91% with GAD diagnosed using the Structured Clinical Interview for the DSM-IV. Women who scored 3 or above were considered to have symptoms consistent with generalized anxiety disorder in the two weeks prior to completing the survey. It is important to note that these tools may only be used to screen for symptoms consistent with PPA or PPD, and are not intended to replace clinical diagnosis.

### Socio-demographic factors

Socio-demographic characteristics, such as age (continuous and by age group), were presented in the analysis. Additional characteristics included education level: “What is the highest certificate, diploma or degree that you have completed?” (measured as “less than high school”, “high school graduate”, or “post-secondary graduate”) and marital status: “What is your marital status?” (measured as “married/common law” or “never married/separated/divorced” or “widowed”).

### Maternal support and sense of belonging

Three concepts are measured in this section: maternal support programs, other maternal support, and sense of belonging to the community. Questions related to maternal support programs included: “During your pregnancy with [your child], did you attend pregnancy support programs? For example, programs where you learned about having a healthy pregnancy or caring for your baby.” (Yes/No); and “Since the birth of your baby, did you attend any parenting support programs? For example, programs where you learn about caring for your child, breastfeeding support, parenting skills and infant or child development” (Yes/No). Other maternal support was measured by the question, “Since the birth of your baby, when you needed other support, how often was it available?” (analyzed as “all or most of the time” or “some, a little or none of the time”). Respondents were also asked “How would you describe your sense of belonging to your local community?” (measured as “strong”, “very strong” or “somewhat weak”, “very weak”), which we refer to as sense of belonging.

### History of depression and treatment

Questions regarding history of depression or a mood disorder, and treatment were also analyzed for this study, including: “Has a doctor, midwife, nurse or other health care worker ever told you that you had depression or a mood disorder?” (measured as “Yes, before you were pregnant with [your child]”, “Yes, while you were pregnant with [your child]”, “Yes, since the birth of [your child]”, “No.”). Women were also asked if they received mental health care: “Since the birth of your baby, have you received treatment for your emotions or mental health?” (measured as “Yes, medication, such as antidepressants”, “Yes, counselling, such as talk therapy”; “Yes, medication and counselling”; “No.”).

### Self-reported mental health, general health, and life satisfaction

Respondents were asked: “Since the birth of your baby, did you ever feel so sad, miserable or anxious that you were concerned about your emotions or mental health?”; “At these times, when you were concerned about your emotions or mental health, did you talk to anyone? [Select all that apply]”; “Was it: 1: Family doctor, midwife, or nurse; 2: Psychiatrist, psychologist, social worker, or counsellor; 3: Spouse or partner; 4: Family or friend; 5: Someone else.”

Respondents were asked “In general, how would you rate your physical health?” and “In general, how would you rate your mental health?” (both measured as “excellent”, “very good” or “good”, “poor” or “fair.”)

In order to assess life satisfaction, respondents were asked: “Using a scale of 0 to 10, where 0 means “Very dissatisfied” and 10 means “Very satisfied”, how do you feel about your life as a whole right now?”

## Analysis

Descriptive analyses were conducted for women with symptoms consistent with PPA and PPD, as well as those who did not experience symptoms consistent with either condition, as a reference category. Weighted proportions, means, and 95% confidence intervals (CI) were presented to provide national estimates. CIs were calculated using the bootstrap method (with 1000 replications), in order to account for the complex sampling design.

Two logistic regression models were used to examine the relationship between PPA and PPD, separately, with socio-demographic factors, maternal support programs, availability of other types of support, sense of belonging, history of depression, and self-rated physical health. Analyses were conducted in SAS EG 5.1 (SAS Institute Inc., Cary, NC, USA). Cases with missing data on a variable were excluded from analyses which used that variable. The primary null hypothesis of our multiple logistic regression models was that there is no relationship between the potential risk factors selected and the incidence of PPA or PPD.

## Results

Overall, 13.8% of women who gave birth in Canada between January 1 and June 30, 2018 had symptoms consistent with PPA, while the prevalence of symptoms consistent with PPD was 17.9%.

### Unadjusted results

Descriptive characteristics of women who had symptoms consistent with PPA, PPD, or neither condition are outlined in Table 1. The largest proportion of women with symptoms consistent with PPA or PPD were in the 25–29 and 30–35 age groups, were married or in a common-law relationship, and had a post-secondary education. These associations were similar to women who did not experience symptoms consistent with either condition.

**Table 1:** Descriptive characteristics of women who had symptoms consistent with PPA, PPD, or neither condition

Variable	PPA			PPD			Neither condition		
	Percent	95% confidence limits		Percent	95% confidence limits		Percent	95% confidence limits	
Age category	*n* = 882*			*n* = 1131*			*n* = 5047		
17–24	10.4	7.7	13.0	10.6	8.2	13.0	7.1	6.2	8.0
25–29	27.9	24.2	31.6	24.8	21.6	28.1	24.0	22.6	25.4
30–34	37.0	32.9	41.0	37.7	34.3	41.1	38.5	36.9	40.1
35–39	19.3	16.2	22.4	21.2	18.4	24.0	23.8	22.3	25.2
40+	5.5	3.8	7.3	5.6	4.0	7.2	6.7	5.8	7.5
Marital status	*n* = 888*			*n* = 1138*			*n* = 5066		
Married/common law	83.1	79.8	86.5	84.5	81.6	87.4	92.0	91.0	93.0
Not married	16.9	13.5	20.2	15.5	12.6	18.4	8.0	7.0	9.0
Education level	*n* = 885*			*n* = 1135*			*n* = 5063		
Less than high school	9.2	6.6	11.8	8.3	6.2	10.5	5.6	4.7	6.4
High school graduate	18.4	15.1	21.6	19.5	16.5	22.4	16.2	14.9	17.5
Post-secondary graduate	72.4	68.7	76.2	72.2	68.9	75.6	78.2	76.8	79.7
Self-reported mental health	*n* = 887*			*n* = 1134*			*n* = 5046		
Poor, fair, or good	76.4	73.0	79.8	82.0	79.1	84.9	30.0	28.4	31.6
Very good or excellent	23.6	20.2	27.0	18.0	15.1	20.9	70.0	68.4	71.6
Self-reported physical health	*n* = 887*			*n* = 1138*			*n* = 5065		
Poor, fair, or good	37.6	36.1	39.1	64.5	61.1	68.0	34.5	33.0	36.1
Very good or excellent	62.4	60.9	63.9	35.5	32.0	38.9	65.5	63.9	67.0
Life satisfaction	*n* = 886*			*n* = 1138*			*n* = 5063		
Low	69.2	65.8	72.6	62.2	58.4	66.0	20.2	18.8	21.6
High	30.8	27.4	34.2	37.8	34.0	41.6	79.8	78.4	81.2
Sense of community belonging	*n* = 881*			*n* = 1134*			*n* = 5049		
Somewhat weak, very weak	44.7	40.6	48.7	46.1	42.4	49.9	20.2	18.9	21.5
Very strong, somewhat strong	55.3	51.3	59.4	53.9	50.1	57.6	79.8	78.5	81.1
Availability of other types of support since birth of baby	*n* = 881*			*n* = 1133*			*n* = 5017		
All/most of the time	48.3	44.1	52.5	53.2	49.5	56.9	35.4	33.9	37.0
Some/little/none of the time	51.7	47.5	55.9	46.8	43.1	50.5	64.6	63.0	66.1
Pregnancy support program	*n* = 888			*n* = 1138			*n* = 5061		
Yes	22.5	19.2	25.9	23.7	20.8	26.6	22.4	21.1	23.8
No	77.5	74.1	80.8	76.3	73.4	79.2	77.6	76.2	78.9
Parenting support program	*n* = 888*			*n* = 1139*			*n* = 5066		
Yes	34.7	30.9	38.6	33.8	30.3	37.4	29.1	27.6	30.6
No	65.3	61.4	69.1	66.2	62.6	69.7	70.9	69.4	72.4
Concerned about mental health	*n* = 888*			*n* = 1139*			*n* = 5066		
Yes	68.2	64.3	72.1	71.6	68.3	74.9	23.0	21.6	24.4
No	31.8	27.9	35.7	28.4	25.1	31.7	77.0	75.6	78.4
Talked to someone	*n* = 623*			*n* = 822*			*n* = 1214		
Yes	81.4	77.6	85.1	81.2	77.9	84.5	89.0	86.9	91.1
No	18.6	14.9	22.4	18.8	15.5	22.1	11.0	8.9	13.1
Family doctor, midwife, or nurse	*n* = 504*			*n* = 658*			*n* = 1066		
Yes	49.9	44.4	55.4	47.9	42.9	52.8	41.4	37.7	45.0
No	50.1	44.6	55.6	52.1	47.2	57.1	58.6	55.0	62.3
Psychiatrist, psychologist, social worker, or counsellor	*n* = 504*			*n* = 658*			*n* = 1066		
Yes	31.2	26.0	36.3	30.6	26.1	35.2	20.5	17.6	23.5
No	68.8	63.7	74.0	69.4	64.8	73.9	79.5	76.5	82.4
Spouse or partner	*n* = 504*			*n* = 658*			*n* = 1066		
Yes	70.5	65.0	75.9	70.0	65.5	74.5	80.6	77.5	83.7
No	29.5	24.1	35.0	30.0	25.5	34.5	19.4	16.3	22.5
Family or friend	*n* = 504			*n* = 658			*n* = 1066		
Yes	72.1	66.9	77.2	71.0	66.6	75.4	75.9	72.9	78.9
No	27.9	22.8	33.1	29.0	24.6	33.4	24.1	21.1	27.1
Someone else	*n* = 504			*n* = 658			*n* = 1066		
Yes	7.7	4.7	10.7	7.9	5.4	10.5	4.6	2.9	6.2
No	92.3	89.3	95.3	92.1	89.5	94.6	95.4	93.8	97.1
History of depression or a mood disorder (ever)	*n* = 885*			*n* = 1136*			*n* = 5068		
Yes	37.1	33.2	41.0	31.7	28.4	35.0	11.2	10.2	12.2
No	62.9	59.0	66.8	68.3	65.0	71.6	88.8	87.8	89.8
Treatment since birth of baby in women with a history of depression	*n* = 330*			*n* = 364*			*n* = 600		
No treatment	40.7	33.9	47.5	41.8	35.3	48.2	55.8	50.9	60.6
Medication	23.7	17.8	29.5	25.2	19.4	31.0	23.6	19.8	27.4
Counselling	17.6	12.2	23.0	18.4	13.4	23.4	9.7	6.8	12.6
Medication and counselling	18.0	13.0	23.0	14.7	10.2	19.2	10.9	7.8	14.0

*Significant chi-square test with *p* < 0.05

More than three quarters of women with symptoms consistent with PPA (76.4%) and 82.0% of women with symptoms consistent with PPD rated their mental health as being “poor”, “fair”, or “very good”, which is considerably higher than those who did not have symptoms consistent with either condition (30.0%). With regard to physical health, 64.5% of women with symptoms consistent with PPD rated their physical health as “poor”, “fair”, or “good”, which is notably higher than women with symptoms consistent with PPA (37.6%) or those who did not have symptoms consistent with either condition (34.5%).

A smaller proportion of women who had symptoms consistent with PPA and PPD, 55.3% and 53.9%, respectively, felt that they had a strong sense of community belonging and had other postpartum support available all or most of the time compared with women who did not have symptoms consistent with either condition (79.8%). Prevalence estimates for the use of pregnancy support programs and parenting support programs were similar in all three groups examined (i.e., PPA, PPD, or neither condition).

Of those who ever had a history of depression or a mood disorder, and who had symptoms consistent with PPD at the time they completed the survey, 41.8% had received no treatment since the birth of their baby; 25.2% had been treated with medication, such as antidepressants; 18.4% had counselling, such as talk therapy; and 14.7% had been treated with both medication and counselling. Similar proportions were reported in women who had symptoms consistent with PPA.

Among women who had symptoms consistent with PPD or PPA, 71.6% and 68.2%, respectively, reported being concerned about their emotions or mental health, which is substantially higher than those who did not have symptoms consistent with either disorder (23.0%). More than eight out of ten women who had symptoms consistent with PPA (81.4%) or PPD (81.2%) and were concerned about their emotions or mental health spoke to someone about it, which is slightly lower than women who did not have symptoms consistent with either condition who had concerns about their emotions or mental health (89.0%).

Of those who spoke to someone and had symptoms consistent with PPD, 47.9% spoke to a family doctor, midwife, or nurse; 30.6% spoke to a psychiatrist, psychologist, social worker, or counsellor; 70.0% spoke to their spouse or partner; 71.0% spoke to their family or a friend; and 7.9% spoke to someone else. These estimates were similar for women who had symptoms consistent with PPA.

The chi-square tests indicated that all variables studied were significantly associated with PPA and PPD, with the exception of pregnancy support programs and talking to “family or friend” or “someone else” when concerned about mental health.

### Adjusted results

The association between socio-demographic factors, social factors, history of depression or a mood disorder, and self-rated physical health and PPA and PPD are presented in Table 2.

**Table 2:** Association between socio-demographic factors, social factors, history of depression or a mood disorder, and self-rated physical health and PPA or PPD

Variable	aOR PPA, 95% CI			aOR PPD, 95% CI		
	Point estimate	95% Wald confidence limits		Point estimate	95% Wald confidence limits	
Age	1.0	1.0	1.0	1.0	1.0	1.0
Education attainment						
Less than high school	0.9	0.7	1.2	1.2	1.0	1.6
High school graduate	1.3	0.9	2.0	1.4	0.9	2.0
Post-secondary graduate	Ref			Ref		
Marital status						
Married/common law	Ref			Ref		
Never married/separated/divorced or widowed	**1.6**	1.2	2.3	**1.5**	1.1	2.1
Attended pregnancy support program						
Yes	Ref			Ref		
No	1.1	0.9	1.4	0.9	0.8	1.1
Attended parent support program						
Yes	1.3	1.1	1.6	1.2	1.0	1.5
No	Ref			Ref		
Availability of other types of support since birth of baby						
All/most of the time	Ref			Ref		
Some/little/none of the time	**1.5**	1.2	1.8	**1.9**	1.6	2.3
Sense of belonging to local community						
Very strong/somewhat strong	Ref			Ref		
Somewhat weak/very weak	**2.1**	1.7	2.5	**2.4**	2.0	2.8
History of depression or a mood disorder (ever)						
Yes	**3.4**	2.7	4.2	**2.6**	2.2	3.2
No	Ref			Ref		
Self-rated physical health						
Excellent/very good	Ref			Ref		
Good/fair/poor	**2.0**	1.7	2.4	**2.4**	2.0	2.9

Bold values represent significant chi-square test with *p* < 0.05

The logistic regression models showed that women who had a history of depression were 3.4 times (95% CI 2.7–4.2) more likely to experience PPA and 2.6 times more likely to experience PPD (95% CI 2.2–3.2) compared with women who did not. Women who reported good, fair, or poor physical health were 2.4 times more likely to experience PPD (95% CI 2.0–2.9) and 2.0 times more likely to experience PPA (95% CI 1.7–2.4) compared with those who reported having very good or excellent health. Women who reported a somewhat or very weak sense of belonging to local community were more likely to experience PPA (aOR 2.1, 95% CI 1.7–2.5) and PPD (aOR 2.4, 95% CI 2.0–2.8) compared with those who reported having a very strong or somewhat strong sense of belonging. Finally, women who were never married, or were separated, divorced, or widowed had significantly higher odds of PPA (aOR 1.6, 95% CI 1.2–2.3) and PPD (aOR 1.5, 95% CI 1.1–2.1) compared with women who were married or common law. Age, education, and attending pregnancy support programs were not significantly associated with PPA and PPD.

## Discussion

We found that approximately one in four women who gave birth in Canada between January 1 and June 30, 2018 reported symptoms consistent with PPA or PPD five to thirteen months postpartum. This is comparable with estimates reported by the United States (US) Centers for Disease Control and Prevention (CDC), where up to one in five women are estimated to experience symptoms of PPD in some states (CDC 2020). Historically, the first national PPD estimates were measured in Canada through the Maternal Experiences Survey (MES) in 2006 (PHAC 2009). The results of this survey indicated that 7.5% of women reported having symptoms consistent with depression during the postpartum period; however, due to differences in survey design, including the use of different screening tools, the results of the SMH are not directly comparable with those of the MES. The higher estimates observed in our study may be explained in part by increased mental health awareness in Canada in the last decade, which may have contributed to the respondents’ ability to identify and report on symptoms specific to PPA or PPD. Further, unlike for the MES, where responses were collected by a human interviewer, responses to the SMH were largely collected through an online questionnaire. This allows for greater disclosure of symptoms of psychological distress than traditional interviewing methods, which involve a human interviewer, and may have contributed, to some extent, to the higher estimates observed (Milton et al. 2017; Duffy et al. 2005).

### Socio-demographic factors

Marital status was a significant predictor of PPA and PPD in this study. Women who were never married, or were separated, divorced, or widowed were more likely to report symptoms consistent with PPA or PPD. While being married or in a relationship may present certain advantages, it is ultimately the quality of the relationship which contributes to a woman’s well-being during the postpartum period, as demonstrated in two meta-analyses (Yim et al. 2015; O’Hara and Swain 1996). The SMH did not collect any information related to the quality of the relationship, which is a limitation of this study.

### History of depression and treatment

Similar to a meta-analysis conducted by Yim et al., having a history of depression was one of the strongest predictors of PPD. This study also showed that among women who had ever been told that they had depression or a mood disorder, the greatest proportion received no treatment, followed by receiving treatment with medication, treatment with counselling, and finally, treatment with both medication and counselling.

Treatment for PPA or PPD varies based on the severity of symptoms and the level of functional impairment. When diagnosed, PPD is considered to be a major depressive disorder, which is typically treated with antidepressants (Sharma and Sharma 2012). Psychotherapy may also have good results either alone or combined with antidepressants for severe depression. For mild or moderate cases of depression or anxiety, the recommended treatment is psychological treatment, or psychosocial strategies, such as peer support or nondirective counselling, and may include medication (Stewart and Vigod 2016). While the SMH did not assess the severity of illness, these recommendations may in part explain the choice in treatment observed in our study. Further, similar to in the general population, availability and accessibility of mental health care services may be a barrier to women receiving adequate care (Henderson et al. 2013).

### Maternal support and sense of belonging

The SMH included several measures of maternal support and belonging. Only the availability of other types of support since the birth of the baby and a strong sense of belonging to local community were associated with lower odds of symptoms consistent with PPA and PPD. These findings are consistent with existing evidence which shows that the benefits of social support can be felt throughout the pregnancy and into motherhood, and may help prevent or mitigate PPD during the antepartum and postpartum period (Li et al. 2017). In particular, a meta-analysis showed that women who were treated with psychosocial interventions were less likely to remain depressed at one-year postpartum than women who received standard postpartum primary care by a health care visitor, public health nurse, or primary care practitioner (Dennis and Hodnett 2007).

### Self-reported mental health, general health, and life satisfaction

Women who had symptoms consistent with PPA or PPD were more concerned about their mental health, had poorer self-reported mental and physical health, and had a lower life satisfaction than those who did not have either condition. These symptoms are often indicative of poor mental health and should be monitored closely in order to identify the onset of illness (Yim et al. 2015; Wilkinson et al. 2017).

Following childbirth, nearly all women report experiencing physical discomfort or pain, primarily in the first eight weeks postpartum. Common symptoms include back pain, perineal pain, pain related to caesarean wound, breast pain, haemorrhoids, constipation, or incontinence (Cooklin et al. 2018). In the SMH, women were surveyed five to thirteen months postpartum, which allows women to recover following the delivery. Therefore, we can speculate that, to some extent, the higher proportion of women with symptoms consistent with PPD observed in this study who reported poor, fair, or good physical health may be a result of physical comorbidities or may reflect respondents including mental health in their global assessments of overall health. This association would benefit from further research.

## Limitations

Due to the nature of the survey, the findings presented in this study are derived from brief screening tools for PPA and PPD, and do not reflect the number of cases of PPA or PPD diagnosed in Canada. In addition, women were surveyed five to thirteen months postpartum, during the winter months. Therefore, we could not examine how responses in the early postpartum months may vary from those six months or longer postpartum, and there could be seasonal effects. The questions asked to screen for symptoms consistent with PPA and PPD were assessed for different time intervals (i.e., symptoms of PPA were assessed over a two-week period, while symptoms of PPD were assessed over a seven-day period); therefore, the two estimates were not to be pooled in order to assess co-occurrence of symptoms. Information on non-respondents was not available, and as such, we cannot assess non-response bias. However, the final sample was weighted to be representative of the Canadian population of women who gave birth during the inclusion period. Finally, this study does not include women in Canada’s territories, where PPA and PPD estimates may differ.

## Conclusion

This study shows that a history of depression and self-reported physical health are most strongly associated with symptoms consistent with PPA or PPD, among the factors that we studied. Protective factors which were significantly associated with symptoms consistent with PPA or PPD were related to marital status and maternal support postpartum, such as the availability of other types of support programs, and a sense of belonging to local community. These findings stress the importance of having adequate maternal support postpartum as a means to support mental health in new mothers. Future studies should aim to determine how to optimize maternal support and to establish adequate timing of delivery of these supports in order to prevent illness.
